# Supplementary material for: Staphylococcus aureus thermonuclease NucA is a key virulence factor in septic arthritis
Source: Commun Biol. 2025 Apr 10;8:598. doi: 10.1038/s42003-025-07920-4 (PMC11986129; doi:10.1038/s42003-025-07920-4)
Supplement: Supplementary file 1 — Supplementary Information [file 42003_2025_7920_MOESM1_ESM.pdf]

Supplementary Information

Supplementary Table 1. Bacterial strains and plasmids used in this study.

Bacterial strains or Plasmids	Descriptions	Reference
E. coli BL21	Laboratory strain	[1]
E. coli DC10B	*E. coli* K12, Δ*dcm* mutant	[2]
S. aureus USA300 JE2	CA-MRSA strain USA300 LAC cured of plasmids	[3]
JE2Δ*nuc1*	*nuc1* gene deleted in JE2	This study
JE2Δ*lgt*	*lgt* gene deleted (phosphatidylglycerol:prolipoprotein diacylglyceryl transferase)	This study
JE2Δ*nuc1*Δ*lgt*	*nuc1-lgt* double deletion	This study
JE2Δ*nuc1*(pRB473-*nuc1*)	pRB473 carrying *nuc1*	This study
S. aureus Newman (NWT)	*S. aureus* Newman wildtype, clinical isolate	[4]
Δ*nuc1*	*nuc1* gene deleted in NWT	This study
Δ*nuc1*(pRB473-*nuc1*)	pRB473 carrying *nuc1*	This study
S. aureus RN4220	Restriction-deficient derivative of NCTC8325-4	[5]
pET28a	Expression plasmid	
pBASE6	Shuttle plasmid	[6]
pRB473	Shuttle plasmid	[7]

Supplementary Table 2. Oligonucleotides used in this study.

Oligonucleotide	Seq
lgtupF	CGCGCAGATCTGTCGACGATGATATGATAAGAAGAGATGTAAGAGTAG
lgtupR	CACTACTTCACTTTTTTTGTGTTAAATACAATACCCATTCAACCTA
lgtdownF	GAATGGGTATTGTATTTAACACAAAAAAAGTGAAGTAGTGATAGTT
lgtdownR	TGCAGGCATGCAAGCTTGATACACGATGATCTTGAACTTCTT
nuc1upF	CGCGCAGATCTGTCGACGATCCATCAACAAATTATACCGTTTTTC
nuc1upR	TGTCTTCGCTCCAAATATTTCATACATATGCCAGCACTTAATA
nuc1downF	TAAGTGCTGGCATATGTATGAAATATTTGGAGCGAAGACAAC
nuc1downR	TGCAGGCATGCAAGCTTGATAACAAGATTACTGAATTATTATGAGATT
nucapET28F	TAAGAAGGAGATATACCATGGCAACTTCAACTAAAAAATTACATAA
nucapET28R	TTCGGGCTTTGTTAGCAGCCTTA TTGACCTGAATCAGCGT
pET28F	GGCTGCTAACAAAGCCCGAAAG
pET28R	CATGGTATATCTCCTTCTTAAAGTTAAACAA
pRB473nuc1F	CGACTCTAGAGGATCAATTTTACAAATAAGGCTAAATA
pRB473nuc1R	GTGCGAATTCGAGCTCTTTTGATACTATTTACTTTTTTAATTCTGAAT

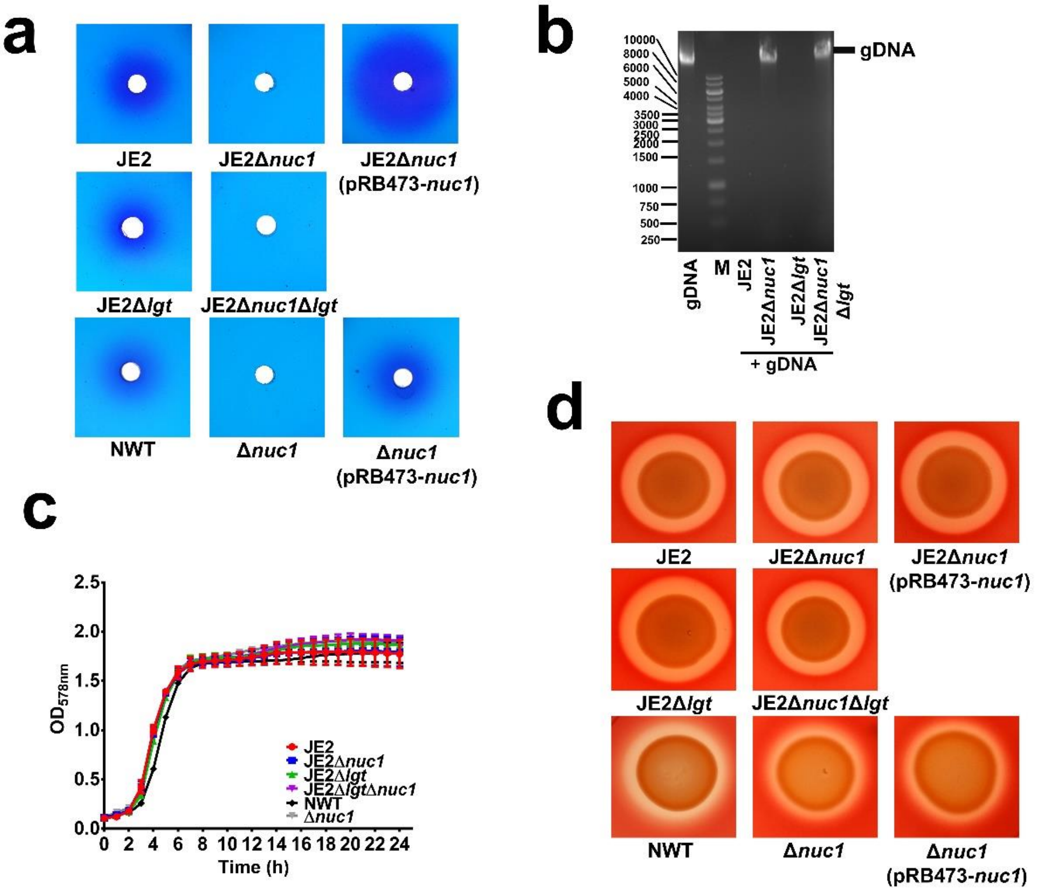

Supplementary Figure 1. Characteristics of JE2, Newman and their mutants. a Nuclease activity, **c** growth in TSB medium, and **d** hemolytic activity of JE2 JE2Δ*nuc1*, JE2Δ*lgt*, JE2Δ*nuc1*Δ*lgt,* JE2Δ*nuc1*(pRB473-*nuc1*), Newman (NWT), Δ*nuc1* and Δ*nuc1*(pRB473-*nuc1*). **b** JE2Δ*lgt* gDNA was incubated with overnight cultural supernatant from JE2, JE2Δ*nuc1*, JE2Δ*lgt*, and JE2Δ*nuc1*Δ*lgt* for 1 h at 37 ^{o}C. The degradation of DNA was visualized through agarose gel electrophoresis.

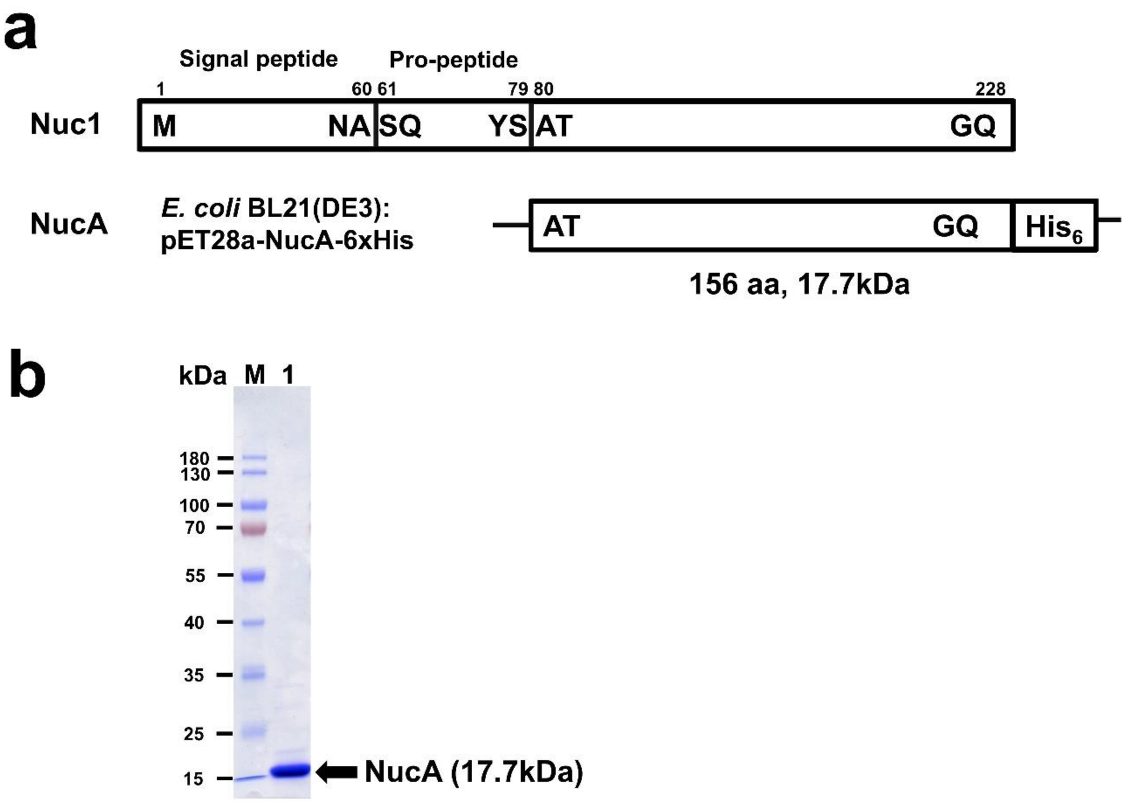

Supplementary Figure 2. Organization of the Nuc1, NucA protein sequence and production of NucA. a Nuc1 is organized as a pre-pro-enzyme. The pre-pro-enzyme is processed to the mature nuclease NucA. Sequence of the mature NucA that was expressed in *E. coli* BL21(DE3) (pET28a-NucA-6x-His) as a C-terminal His-tag fusion protein. **b** Demonstration of Ni-NTA purified NucA in SDS-PAGE. NucA was used in gDNA hydrolysis assays.

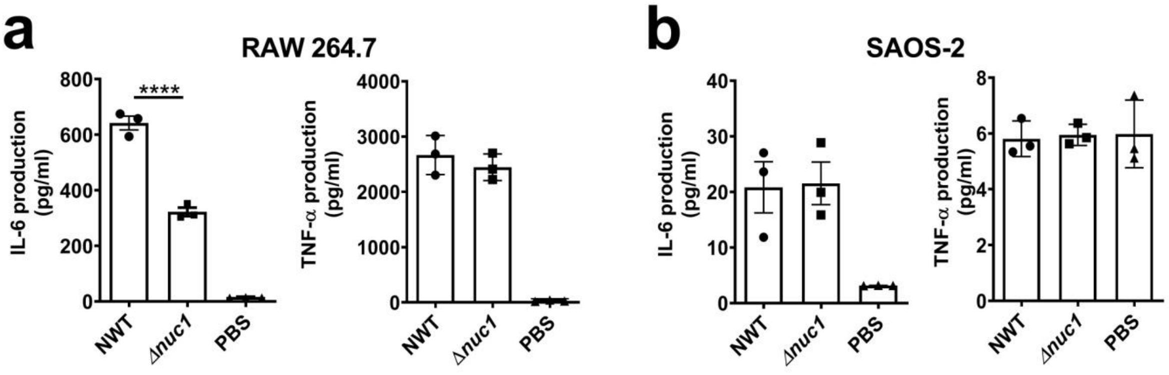

Supplementary Figure 3. Induction of cytokines by host cells upon exposure to live Newman (NWT) and its mutant Δ*nuc1*. The PBS-washed bacteria were incubated with **a** RAW 264.7 at an MOI=30 and **b** SAOS-2 cells at an MOI=3. Cellular supernatants were collected after 18 h for ELISA assay. Triplet experiments were conducted; error bars indicate ± SEM; not significant p>0.05; ****p<0.0001, one-way ANOVA with Dunnett's posttest.

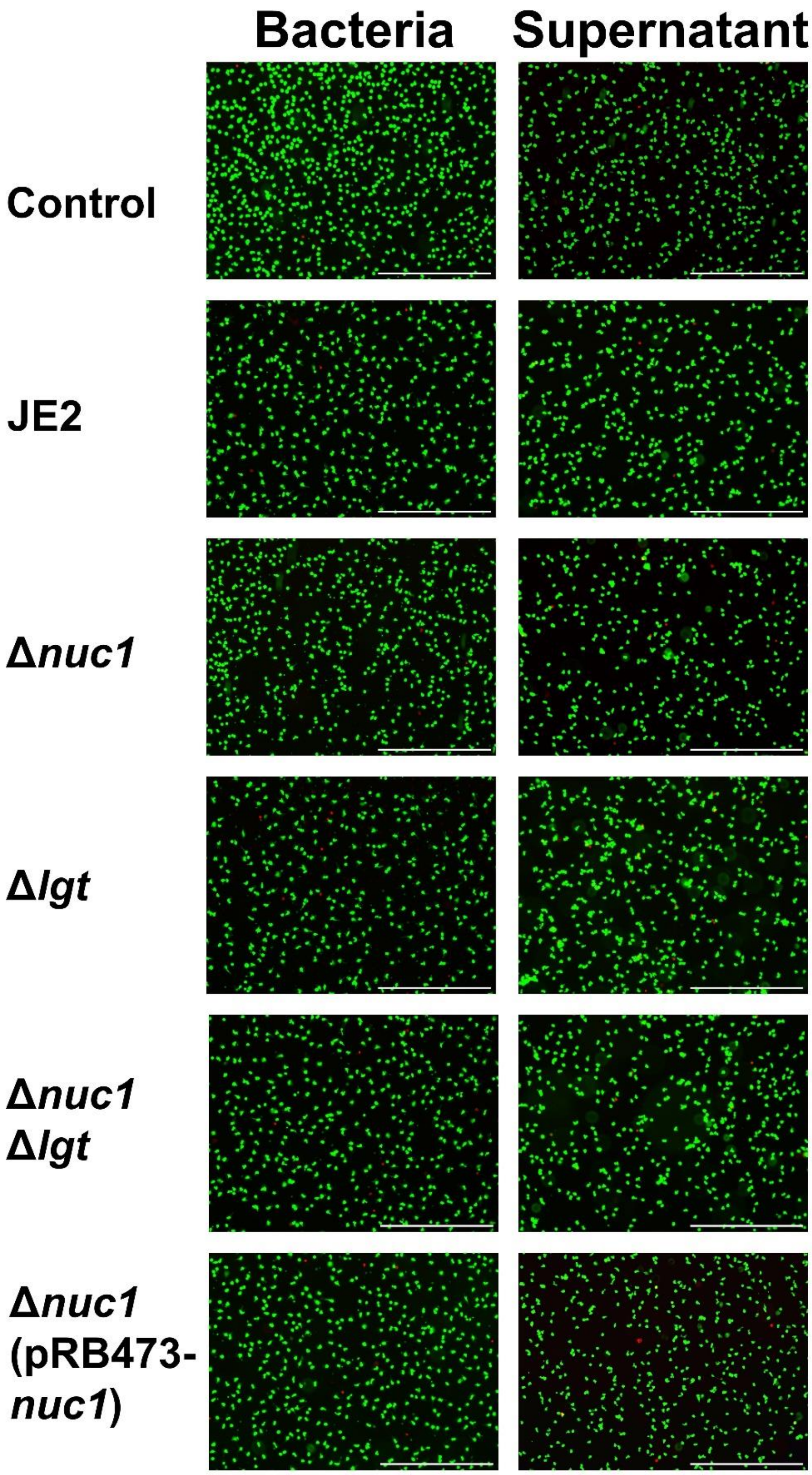

Supplementary Figure 4. Exemplary pictures of viability staining for neutrophils exposed to JE2 and its mutants. Pictures were taken after 3 hours of incubation. Green: Calcein AM, Red: Ethidium Bromide, scale bar: 500 µm.

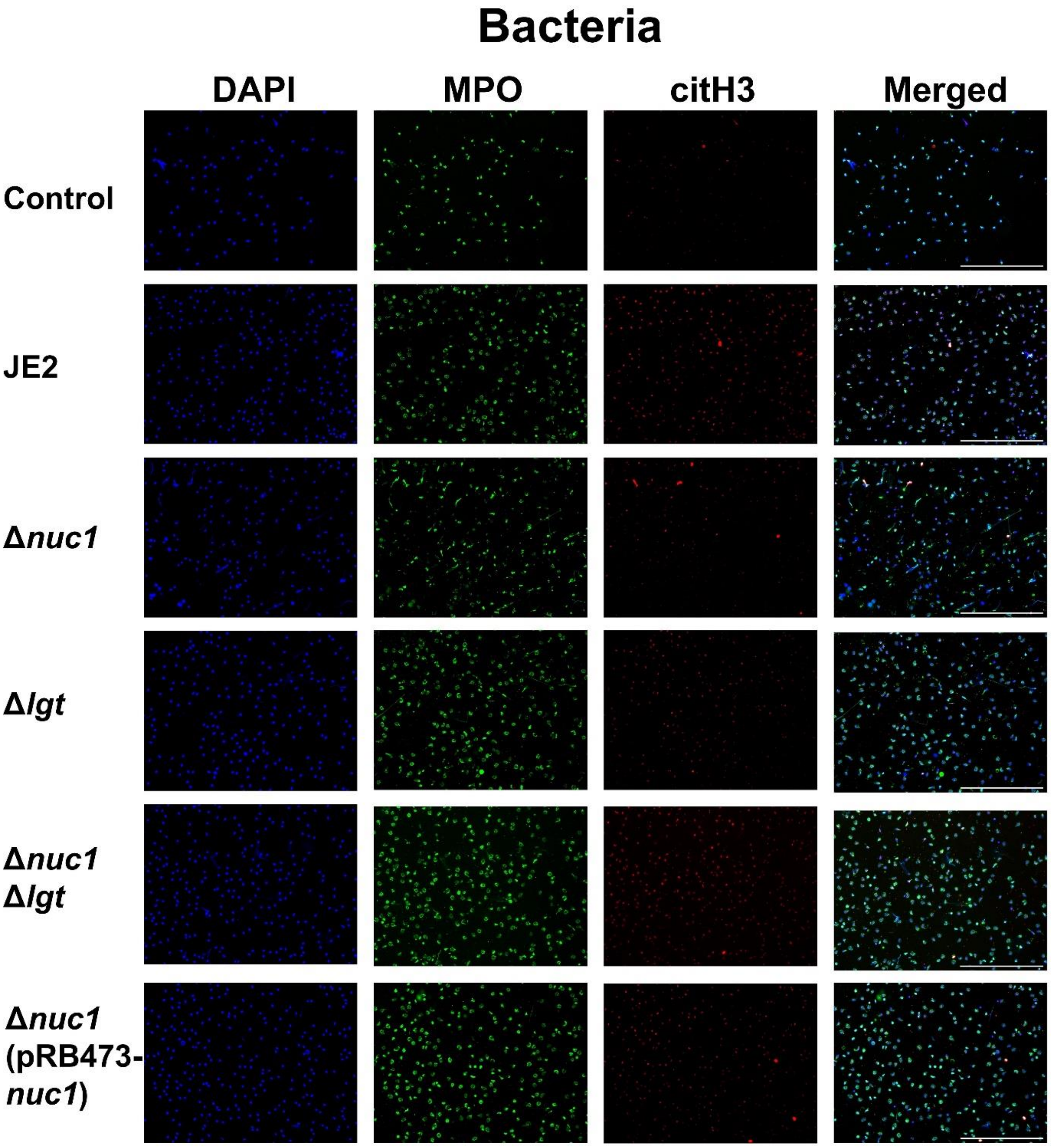

Supplementary Figure 5. Exemplary images of immunofluorescent staining of live bacteria (MOI=2) incubated with neutrophils at 1 h incubation. Blue: DNA (Hoechst 33342); Green: myeloperoxidase, MPO; Red: citrullinated histone H3, citH3, scale bar: 500 µm.

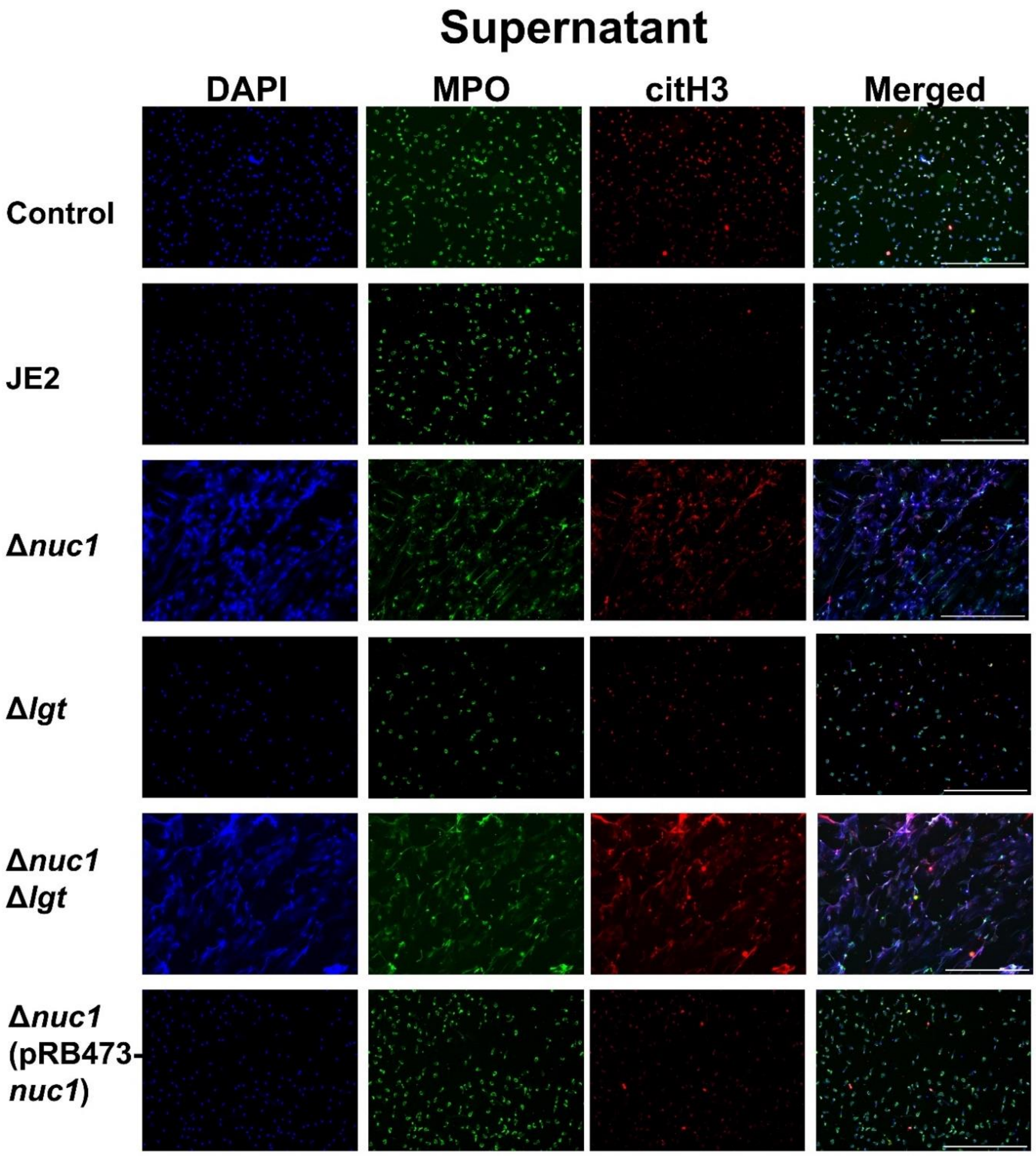

Supplementary Figure 6. **Exemplary images of immunofluorescent staining of overnight supernatant (2% volume) incubated with neutrophils at 1 h incubation.** Blue: DNA (Hoechst 33342); Green: myeloperoxidase, MPO; Red: citrullinated histone H3, citH3, scale bar: 500 µm.

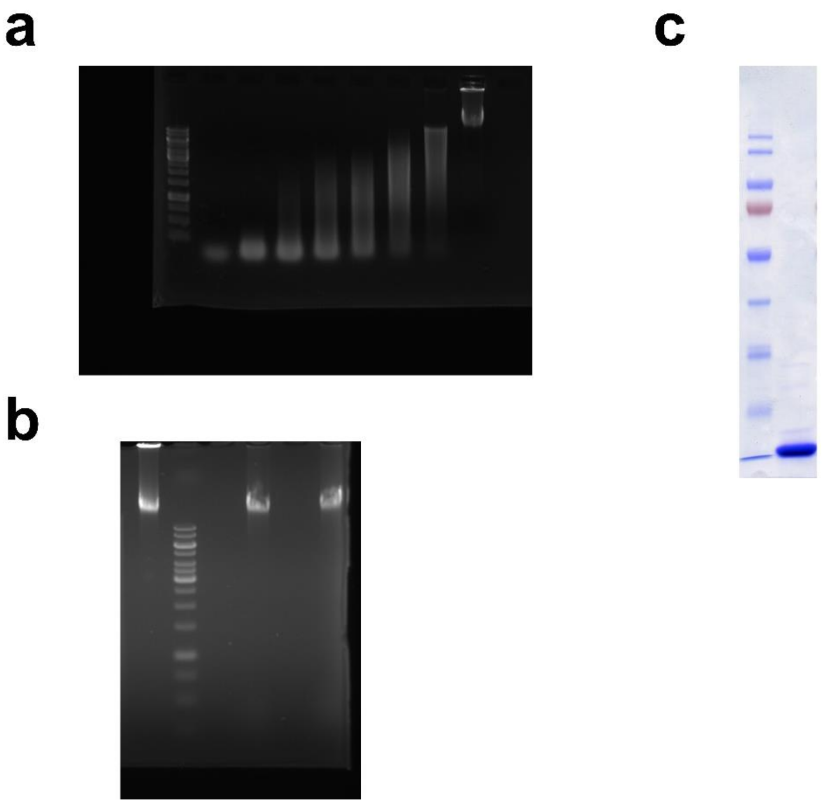

Supplementary Figure 7. **Unedited agarose gel or SDS-PAGE gel images. a** Unedited image for Fig. 3b. **b** Unedited agarose gel image for Supplementary Figure 1b. **c** Unedited SDS-PAGE gel image for Supplementary Figure 2b.

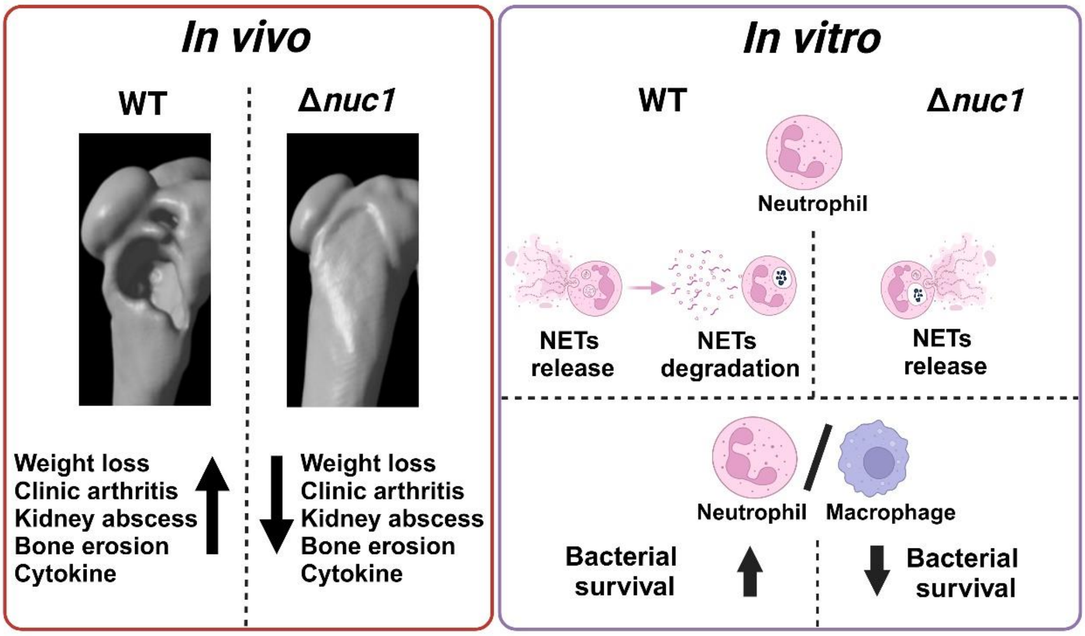

Supplementary Figure 8 (Graphical Abstract). Schematic representation of the differences in pathogenicity between the *S. aureus* wild-type strain (WT) and its Δ*nuc1* mutant in the mouse model for septic arthritis and under *in vitro* conditions. *In vivo*, WT-infected mice showed marked weight loss, increased clinical arthritis frequency, higher kidney abscess score, severe bone erosions, and higher cytokine levels than the Δ*nuc1*-infected mice. *In vitro*, the WT effectively digests NETs formed by neutrophils, which increases bacterial survival. This image is created in BioRender. Götz, F. (2025) https://BioRender.com/w43z906

Supplementary References

1. Studier, F. W. & Moffatt, B. A. Use of bacteriophage T7 RNA polymerase to direct selective high-level expression of cloned genes. *J Mol Biol* **189**, 113-130 (1986).
2. Monk, I. R., Shah, I. M., Xu, M., Tan, M. W. & Foster, T. J. Transforming the untransformable: application of direct transformation to manipulate genetically *Staphylococcus aureus* and *Staphylococcus epidermidis*. *mBio* **3** (2012).
3. Fey, P. D. *et al.* A genetic resource for rapid and comprehensive phenotype screening of nonessential *Staphylococcus aureus* genes. *mBio* **4**, e00537-00512 (2013).
4. Duthie, E. S. & Lorenz, L. L. Staphylococcal coagulase; mode of action and antigenicity. *J Gen Microbiol* **6**, 95-107 (1952).
5. Kreiswirth, B. N. *et al.* The toxic shock syndrome exotoxin structural gene is not detectably transmitted by a prophage. *Nature* **305**, 709-712 (1983).
6. Geiger, T. *et al.* The stringent response of *Staphylococcus aureus* and its impact on survival after phagocytosis through the induction of intracellular PSMs expression. *PLoS Pathog* **8**, e1003016 (2012).
7. Bruckner, R. A series of shuttle vectors for *Bacillus subtilis* and *Escherichia coli*. *Gene* **122**, 187-192 (1992).
