# Supplementary material for: Staphylococcus aureus thermonuclease NucA is a key virulence factor in septic arthritis
Source: Commun Biol. 2025 Apr 10;8:598. doi: 10.1038/s42003-025-07920-4 (PMC11986129; doi:10.1038/s42003-025-07920-4)
Supplement: Supplementary file 4 — Reporting summary [file 42003_2025_7920_MOESM4_ESM.pdf]

nature portfolio

Corresponding author(s): Friedrich Götz

Last updated by author(s): Feb 27, 2025

Reporting Summary

Nature Portfolio wishes to improve the reproducibility of the work that we publish. This form provides structure for consistency and transparency in reporting. For further information on Nature Portfolio policies, see our Editorial Policies and the Editorial Policy Checklist.

Statistics

For all statistical analyses, confirm that the following items are present in the figure legend, table legend, main text, or Methods section.

n/a	Confirmed	
☐	☒	The exact sample size (*n*) for each experimental group/condition, given as a discrete number and unit of measurement
☐	☒	A statement on whether measurements were taken from distinct samples or whether the same sample was measured repeatedly
☐	☒	The statistical test(s) used AND whether they are one- or two-sided<br>*Only common tests should be described solely by name; describe more complex techniques in the Methods section.*
☒	☐	A description of all covariates tested
☐	☒	A description of any assumptions or corrections, such as tests of normality and adjustment for multiple comparisons
☐	☒	A full description of the statistical parameters including central tendency (e.g. means) or other basic estimates (e.g. regression coefficient) AND variation (e.g. standard deviation) or associated estimates of uncertainty (e.g. confidence intervals)
☐	☒	For null hypothesis testing, the test statistic (e.g. *F*, *t*, *r*) with confidence intervals, effect sizes, degrees of freedom and *P* value noted<br>*Give P values as exact values whenever suitable.*
☒	☐	For Bayesian analysis, information on the choice of priors and Markov chain Monte Carlo settings
☒	☐	For hierarchical and complex designs, identification of the appropriate level for tests and full reporting of outcomes
☒	☐	Estimates of effect sizes (e.g. Cohen's *d*, Pearson's *r*), indicating how they were calculated

Our web collection on statistics for biologists contains articles on many of the points above.

Software and code

Policy information about availability of computer code

Data collection	*Provide a description of all commercial, open source and custom code used to collect the data in this study, specifying the version used OR state that no software was used.*
Data analysis	GraphPad Prism (version 6.0, GraphPad Software)

For manuscripts utilizing custom algorithms or software that are central to the research but not yet described in published literature, software must be made available to editors and reviewers. We strongly encourage code deposition in a community repository (e.g. GitHub). See the Nature Portfolio guidelines for submitting code & software for further information.

Data

Policy information about availability of data

All manuscripts must include a data availability statement. This statement should provide the following information, where applicable:

- Accession codes, unique identifiers, or web links for publicly available datasets
- A description of any restrictions on data availability
- For clinical datasets or third party data, please ensure that the statement adheres to our policy

All the data in this study are available from the corresponding author upon reasonable request.

Research involving human participants, their data, or biological material

Policy information about studies with human participants or human data. See also policy information about sex, gender (identity/presentation), and sexual orientation and race, ethnicity and racism.

Reporting on sex and gender	The average age of the volunteers was 26.8 ± 3.9 years, with half being female and half male.
Reporting on race, ethnicity, or other socially relevant groupings	N/A
Population characteristics	N/A
Recruitment	Blood and serum were collected from healthy adult volunteers and written informed consent was given.
Ethics oversight	This study was done in accordance with the ethics committee of the medical faculty of the University of Tübingen that approved the study (Approval number 015/2014 BO2).

Note that full information on the approval of the study protocol must also be provided in the manuscript.

Field-specific reporting

Please select the one below that is the best fit for your research. If you are not sure, read the appropriate sections before making your selection.

☒ Life sciences ☐ Behavioural & social sciences ☐ Ecological, evolutionary & environmental sciences

For a reference copy of the document with all sections, see nature.com/documents/nr-reporting-summary-flat.pdf

Life sciences study design

All studies must disclose on these points even when the disclosure is negative.

Sample size	The sample size was performed based on previous work. We took methods in vitro experiments at least n=3, and mouse model with n=5.
Data exclusions	No data was excluded.
Replication	All data were replicated at least n=3 independent data replicates. The source data were provided in Supplementary Data 1.
Randomization	Random number methods was used for mice.
Blinding	When performing the human and animal experimens, it was conducted in a blinded manner to the operators.

Reporting for specific materials, systems and methods

We require information from authors about some types of materials, experimental systems and methods used in many studies. Here, indicate whether each material, system or method listed is relevant to your study. If you are not sure if a list item applies to your research, read the appropriate section before selecting a response.

Materials & experimental systems

n/a	Involved in the study	
☐	☒	Antibodies
☐	☒	Eukaryotic cell lines
☒	☐	Palaeontology and archaeology
☐	☒	Animals and other organisms
☒	☐	Clinical data
☒	☐	Dual use research of concern
☒	☐	Plants

Methods

n/a	Involved in the study	
☒	☐	ChIP-seq
☒	☐	Flow cytometry
☒	☐	MRI-based neuroimaging

Antibodies

Antibodies used	myeloperoxidase (sc-52707, Santa Cruz Biotechnology)<br>citH3 (ab5103, Abcam)<br>Alexa Fluor-488 conjugated secondary antibody (A10667, Invitrogen)

Validation	All antibodies used in this study were commercially available and used for immunofluorescence staining. They were validated by the manufacturers and previous publications.

Eukaryotic cell lines

Policy information about cell lines and Sex and Gender in Research

Cell line source(s)	The RAW264.7 cell line was purchased from ATCC. The SAOS-2 cell line was provided by Stefanie Krajewski, who also purchased from ATCC.
Authentication	Cells were verfiried by manufacturers's website. Previous studies have shown their ability in immune response and immune signaling pathway.
Mycoplasma contamination	No mycoplasma contamination. The cells were tested by Mycoplasma Detection Kit (Invitrogen).
Commonly misidentified lines (See ICLAC register)	No commonly misidentified cell lines were used in this study.

Animals and other research organisms

Policy information about studies involving animals; ARRIVE guidelines recommended for reporting animal research, and Sex and Gender in Research

Laboratory animals	Naval Medical Research Institute (NMRI) mice female mice, aged 8 weeks, were purchased from Envigo (Venray, Netherlands).
Wild animals	No wild animals were used in this study.
Reporting on sex	Female mice were used for this study.
Field-collected samples	This study did not involve samples collected from field
Ethics oversight	The Ethics Committee of Animal Research of Gothenburg approved all experiments conducted on mice. The mouse experiments were performed in accordance with the Swedish Board of Agriculture's regulations and recommendations on animal experiments.

Note that full information on the approval of the study protocol must also be provided in the manuscript.

Plants

Seed stocks	*Report on the source of all seed stocks or other plant material used. If applicable, state the seed stock centre and catalogue number. If plant specimens were collected from the field, describe the collection location, date and sampling procedures.*
Novel plant genotypes	*Describe the methods by which all novel plant genotypes were produced. This includes those generated by transgenic approaches, gene editing, chemical/radiation-based mutagenesis and hybridization. For transgenic lines, describe the transformation method, the number of independent lines analyzed and the generation upon which experiments were performed. For gene-edited lines, describe the editor used, the endogenous sequence targeted for editing, the targeting guide RNA sequence (if applicable) and how the editor was applied.*
Authentication	*Describe any authentication procedures for each seed stock used or novel genotype generated. Describe any experiments used to assess the effect of a mutation and, where applicable, how potential secondary effects (e.g. second site T-DNA insertions, mosiacism, off-target gene editing) were examined.*
